# How effective are volunteers at supporting people in their last year of life? A pragmatic randomised wait-list trial in palliative care (ELSA)

**DOI:** 10.1186/s12916-016-0746-8

**Published:** 2016-12-09

**Authors:** Catherine Walshe, Steven Dodd, Matt Hill, Nick Ockenden, Sheila Payne, Nancy Preston, Guillermo Perez Algorta

**Affiliations:** 1 International Observatory on End of Life Care, Division of Health Research, Lancaster University, Bailrigg, Lancaster LA1 4YW UK; 2 Division of Health Research, Lancaster University, Bailrigg, Lancaster LA1 4YW UK; 3 Institute for Volunteering Research, NCVO, Society Building, 8 All Saints Street, London, N1 9RL UK

**Keywords:** Pragmatic clinical trial, Randomised controlled trial, Volunteers, Palliative care

## Abstract

**Background:**

Clinical care alone at the end of life is unlikely to meet all needs. Volunteers are a key resource, acceptable to patients, but there is no evidence on care outcomes. This study aimed to determine whether support from a social action volunteer service is better than usual care at improving quality of life for adults in the last year of life.

**Methods:**

A pragmatic, multi-centre wait-list controlled trial, with participants randomly allocated to receive the volunteer support intervention either immediately or after a 4 week wait. Trained volunteers provided tailored face-to-face support including befriending, practical support and signposting to services, primarily provided within the home, typically for 2–3 hours per week. The primary outcome was rate of change of quality of life at 4 weeks (WHO QOL BREF, a general, culturally sensitive measure). Secondary outcomes included rate of change of quality of life at 8 weeks and Loneliness (De Jong Gierveld Loneliness Scale), social support (mMOS-SS), and reported use of health and social care services at 4 and 8 weeks.

**Results:**

In total, 196 adults (61% (*n* = 109) female; mean age 72 years) were included in the study. No significant difference was found in main or secondary outcomes at 4 weeks. Rate of change of quality of life showed trends in favour of the intervention (physical quality of life domain: *b* = 3.98, CI, –0.38 to 8.34; psychological domain: *b* = 2.59, CI, –2.24 to 7.43; environmental domain: *b* = 3, CI, –4.13 to 4.91). Adjusted analyses to control for hours of volunteer input found significantly less decrease in physical quality of life in the intervention group (slope (*b*) 4.43, CI, 0.10 to 8.76). While the intervention also favoured the rate of change of emotional (*b* = –0.08; CI, –0.52 to 0.35) and social loneliness (*b* = –0.20; CI, –0.58 to 0.18), social support (*b* = 0.13; CI, –0.13 to 0.39), and reported use of health and social care professionals (*b* = 0.16; CI, –0.22 to 0.55), these were not statistically significant. No adverse events were reported.

**Conclusions:**

Clinicians can confidently refer to volunteer services at the end of life. Future research should focus on ‘dose’ to maximise likely impact.

**Trial registration:**

The trial was prospectively registered. ISRCTN Registry: ISRCTN12929812, registered 20 May 2015.

## Background

In 2013, over half a million people died in England and Wales, mostly from long-term conditions such as cancer (29%) and circulatory (28%) and respiratory (15%) diseases that are known to be life limiting [1]. For deaths that can be anticipated, providing excellent care at the end-of-life that is responsive to need is critically important. Compassionate support in the last year of life cannot be the responsibility of health and social care professionals alone and requires a public health response involving the wider community [2, 3], recognising the importance of social networks and social capital [4]. Proponents of these approaches argue that a primary focus on biomedical and physical aspects of end-of-life care ignores the social context within which dying takes place. Social relationships and networks can buffer the effects of crisis associated with dying, provide a framework that may prevent family carer burn out, and demonstrate the importance of supporting social contexts [5–7]. Effective personal network support can substitute for formal care and reduce health service utilisation costs [8].

Individual and community networks and relations of support can, however, be inadequate to meet care needs [5]. Demographic changes such as increased female employment, delayed childbearing, geographical mobility, divorce rates, and longer working lives all potentially impact on the availability of traditional family support. Social isolation on itself also has a major influence on health, comparable with well-established risk factors for mortality [9].

To supplement both clinical and community care many services are using volunteers as a critical part of the multi-disciplinary care offered at the end of life [10, 11]. Volunteers are important to care, and indeed 42% of the adult population volunteer formally [12], with an estimated 3 million in health and social care [13] and 125,000 within hospices [14]. The Department of Health commitment to end-of-life care specifically recognises that such care is not simply ‘medical issues with medical solutions’, and pledges to developing the work of end-of-life care volunteer networks [15].

It is known that people are happy with volunteer support at the end of life [16–20], but there is little evidence of their effect on care outcomes. Evaluation in well-designed comparative studies is therefore recommended [11]. This is particularly apt in palliative and end-of-life care, where the effectiveness of many interventions are not evaluated using robust designs; this is potentially wasteful of resources and could lead to poorer or unintended outcomes. This is the first randomised trial of volunteer delivered support services at the end of life to evaluate the effectiveness of such interventions.

## Methods

### Design

This study was a pragmatic, randomised, prospective open wait-list trial. The protocol for the study is published [21]. The trial used a wait-list design to randomly allocate participants on a 1:1 basis to receive the intervention either immediately or after a 4 week wait [22–24]. A wait-list approach, where consented participants are allocated to either receive an intervention immediately or after a defined period on a waiting list during which they receive usual care is regarded as more ethically defensible in end-of-life care, and allows a valid comparison between the experimental and control arms at the time when the delayed intervention starts [23–27].

### Participants and setting

Participants in the trial include people anticipated to be in their last year of life and their self-identified informal carer. Very low numbers of carers were recruited and there is insufficient data to report. Inclusion criteria were broad to include typical participants of such services.

Patient inclusion criteria:

1. Those eligible to be referred to an end-of-life care service determined by the referring organisation/individual. They should be able to answer ‘no’ to the ‘surprise question’: ‘Would you be surprised if the patient dies within a year?’
2. Able to give informed consent.

Patient exclusion criteria:

1. Age < 18 years.
2. Those who only understand or speak a language in which our main outcome measure (the World Health Organization Quality of Life Brief Scale (WHOQOL-BREF)) is unavailable.
3. Those with an anticipated prognosis of < 4 weeks.

### Setting

Eleven English providers of end-of-life home care services (nine hospices, one alcohol and substance use charity, one NHS Trust), funded to provide the intervention through competitive tender with the UK Cabinet Office. The intervention was provided in community settings, primarily in participant’s own homes.

### Intervention

Volunteers provided face-to-face individual support to people anticipated to be in their last year of life. Volunteer-provided services are a mode of support hypothesised to be distinctive from, but supplementary to, usual forms of health and social care, with an effect on domains of quality of life, loneliness and social support. In this pragmatic trial services had flexibility to deliver the intervention in a locally responsive manner within agreed parameters. Key elements of the volunteer-provided support intervention include its delivery by trained volunteers who were matched to individuals by a volunteer co-ordinator, and provided care tailored to the needs of the individual but offered from a suite of options including befriending, practical support and signposting, which could differ at each visit. Volunteer support was typically provided face-to-face, one-to-one, in the home, but telephone contact and meeting outside the home were possible. Most contacts were befriending visits in the home. The frequency and length of contact was individually determined according to negotiated participant preference and service availability, but was typically a visit once a week for 1–3 hours. The service could continue as required after the study, but study participants were followed up for 8 weeks post intervention commencement. Volunteers could be any age (18+), sex or ethnicity, and all received basic training on boundaries, communication skills and organisational policies according to the requirements of the organisation providing the intervention at each site. Data captured the type, length and frequency of each contact between participant and volunteer.

Participants continued to receive all usual care during the study.

### Objectives

The primary aim of the study was to evaluate the effectiveness of receiving care from a social action volunteer befriending service plus usual care at 4 weeks at improving quality of life compared to usual care alone for adults in the last year of life.

The secondary aims were to:

- Explore whether the social action volunteer befriending service affects quality of life at 8 weeks.
- Explore whether the social action volunteer befriending service reduces loneliness and affects the perception of social support for adults at 4 and 8 weeks.
- Examine whether informal carers for those receiving care from a social action volunteer befriending service experience less carer burden at 4 and 8 weeks.
- Determine whether receiving care from a social action volunteer befriending service can affect participant’s use of other health and social care services at 4 and 8 weeks.

Trial outcome measures:

- Quality of life: WHOQOL-BREF Scale, a short (26 item) non-disease-specific, validated measure of quality of life and wellbeing, having wide breadth and available in many languages [28]. Data are reported across the physical, psychological, environment and social relationship domains which were considered likely to be important outcomes for this intervention. Cronbach’s Alpha (α) (Physical 0.714, Psychological 0.776, Environment 0.712, Social Relationships 0.461). The low Cronbach’s Alpha for social relationships reflects that this is a 3-item subscale, with an item which had many missing values in this study.
- Loneliness: De Jong Gierveld 6-item Loneliness Scale, a short, well-used, reliable and valid measurement instrument for overall (α 0.374), emotional (α 0.678), and social loneliness (α 0.846) [29].
- Social Support: 8-item modified Medical Outcomes Study Social Support Survey (mMOS-SS), a short validated scale covering two domains (emotional (α 0.859) and instrumental (α 0.892) social support, total α 0.910) designed to identify potentially modifiable social support deficits [30].
- Self-reported contact with health and social care services over the previous 2 weeks.

The primary outcome was rate of change of quality of life at 4 weeks. Secondary outcomes were the rate of change of quality of life at 8 weeks, and loneliness, social support and reported use of health and social care services at 4 and 8 weeks. A short time period was chosen for the primary outcome as any end-of-life care intervention needs to work rapidly to be worthwhile. No data were available to predetermine intervention length, but follow-up for 8 weeks facilitates understanding of any persistence of effect.

Socio-demographic data (age, sex, disease diagnosis, education, marital status, living status, spirituality and ethnicity) in the form of a self-completed questionnaire was collected from both patients and informal carers at baseline. At baseline and subsequent time points, patient participants were asked to indicate the number, type and frequency of contact they have with networks of others.

The schedule of data collection is presented in Table 1. Time periods were necessarily short to reflect the possible prognosis of participants.

**Table 1:**
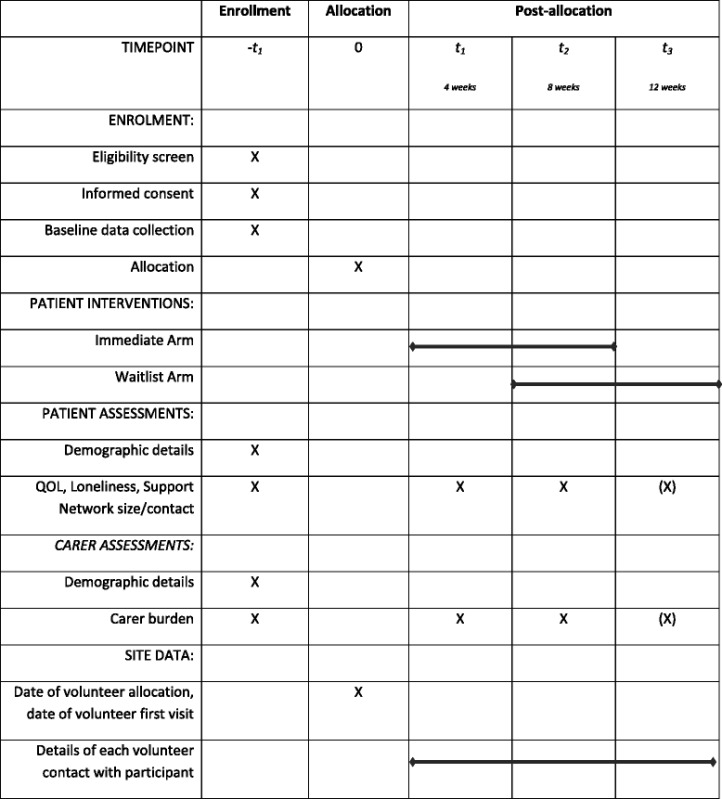
End of Life Social Action Study (ELSA): Schedule of enrolment, interventions and assessments

(X) indicates that week 12 data are only collected for those in the wait-list arm of the trial (8 weeks after commencement of intervention)

### Sample size

Trial power was estimated using a worst case scenario assuming 5% attrition at primary outcome measure. With 350 or more participants per arm power exceeded 0.80 to detect difference in change over time corresponding to an effect size of f = 0.10 between the intervention and wait-list groups. This power model uses alpha = 0.05, two tailed, and uses a conservative correlation of r = 0.6 for scores lagged 4 weeks, and r = 0.5 for 12 weeks.

### Randomisation and study procedures

Participants were those referred to participating services following eligibility, information and informed consent procedures. Baseline data were collected and participants randomly allocated (1:1 allocation ratio) to either the intervention or the wait-list arm of the trial. Site coordinators contacted a randomisation line at Lancaster University, and the next sequence in the allocation (stored in sequentially numbered sealed opaque envelopes) was revealed. The randomisation sequence was computer generated, with rebalance in the arms after 10 randomisations. Blinding of site staff and patient participants was not possible due to the nature of the intervention. Data collected at 4, 8 and 12 weeks were coordinated by the research team and sent by post to patient and carer participants for self-completion. Data were returned directly to the research team.

### Statistical analysis

All analyses were conducted using SPSS version 22. The primary outcome was rate of change of quality of life at 4 weeks (WHOQOL-BREF). Basic exploratory and descriptive statistical tests (e.g. *t* and χ^2^ tests) were conducted at the α = 0.05 individual level of significance. Confidence intervals were reported at the 95% level. Hierarchical Linear Models (HLM) used the full intention-to-treat sample over all available assessments with the aim of differentiating treatment effects from natural trajectories. The HLMs compared primary and secondary outcome scores (e.g. WHOQOL-BREF) between the immediate and wait-list groups. A piecewise model was specified to index change from baseline to week 4 (Phase 1), and change from week 4 to week 12 (Phase 2). Restricted maximum likelihood estimation was used, and fixed slopes and random effects of each time predictor were assessed to determine the most appropriate model. Final models were specified with intercepts as random effects to account for correlations among observations at different time points from the same participant. Treatment condition, time and interactions between treatment condition × time were specified as fixed effects.

With the interaction term, we tested whether there was a significant difference in rate of change between treatment groups before and after week 4. Secondary analyses were conducted by testing the same HLMs described with treatment completers. In the immediate arm, treatment completers were those who received any intervention before 4 weeks, and who returned baseline and week 4 data. In the wait-list arm, treatment completers were those who did not receive any intervention before the return of week 4 data. Finally, sensitivity analyses on primary outcomes were conducted to consider of the amount of volunteer input people received in the initial 4-week period. Models were therefore specified using hours of input and controlling for site.

## Results

### Recruitment and flow through trial

**Fig. 1:**
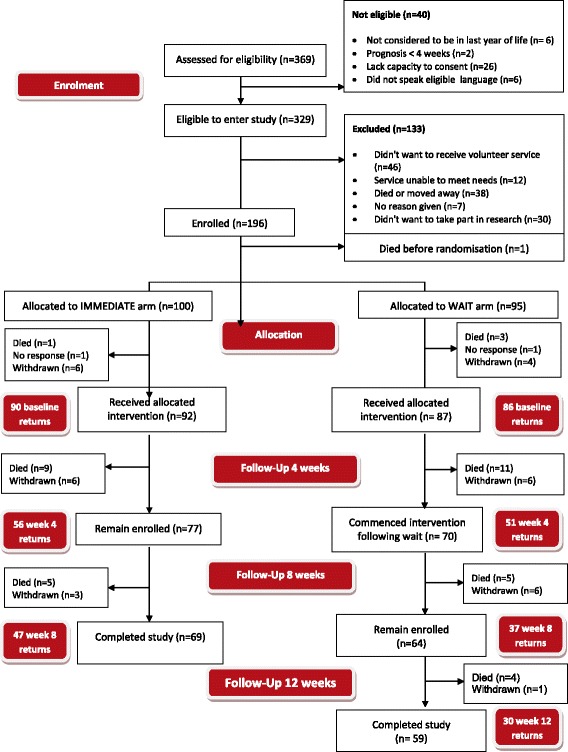
CONSORT diagram of participant flow through the study

Figure 1 outlines the flow of participants in the trial. Recruitment took place from June 2015 to January 2016, with data collection to March 2016. Of 329 eligible people approached to participate, 196 consented. Reasons for the 133 not participating included not wishing to receive the service (*n* = 46), they died or moved away (*n* = 38), or they did not wish to take part in research (*n* = 30). One participant died between consent procedures and randomisation, thus 195 were randomised, 100 to receive the intervention immediately and 95 to the wait-list control group. At each time point, missing data were noted, but participants continued to be enrolled in the study unless advised otherwise, as data sets could be and often were returned at subsequent time points. Around 40% of those in the immediate arm received no volunteer intervention before week 4 assessment due to lack of volunteer availability and matching, and some in the wait-list arm received the intervention at or after 4 weeks, but prior to completing their week 4 assessment. Few carers (n = 33) entered the study and hence their data are not given here. A total of 20% of enrolled participants (39 of 196) died during the study.

### Baseline characteristics

Participants were recruited from all 11 sites (range 3–40 participants per site). Participant mean age was 72 (range 37–92), and 60% were female. No significant differences on demographic aspects between randomised groups were observed (Table 2). No adverse events were reported.

**Table 2:** Baseline and demographic data. Values are numbers (percentages) unless specified otherwise

Demographics	Immediate *n* = 92	Wait *n* = 87
Age, mean ± SD	72 ± 12.03	72 ± 12.50
Sex, female n (%)	56 (61)	53 (61)
Education, standard n (%)	62 (76)	54 (70)
Marital status, single n (%)	54 (61)	61 (72)
Living status, living alone n (%)	47 (53)	54 (64)
Occupation, retired n (%)	74 (86)	70 (82)
Ethnicity, white British n (%)	81 (92)	76 (89)
Spirituality, religious n (%)	58 (71)	51 (69)
Cancer diagnosis, n (%)	37 (41)	47 (55)
Baseline quality of life		
Quality of life, poor or very poor n (%)	38 (44)	37 (44)
Are you dissatisfied with your health? n (%)	62 (70)	65 (76)
Quality of life, mean ± SD		
QoL Physical	32.09 ± 15.21	34.95 ± 17.42
QoL Psychological	46.52 ± 19.10	45.74 ± 17.01
QoL Environment	58.75 ± 16.23	57.05 ± 14.76
QoL Social relationships	55.47 ± 23.26	52.88 ± 26.41
Loneliness, mean ± SD		
Social loneliness	1.51 ± 1.21	1.69 ± 1.25
Emotional loneliness	1.70 ± 1.11	2.12 ± 0.87
Total loneliness score	3.17 ± 1.89	3.77 ± 1.66
Social support, mean ± SD		
mMOSS instrumental	3.27 ± 1.31	3.00 ± 1.28
mMOSS emotional	3.25 ± 1.10	3.98 ± 1.09
mMOSS total	3.27 ± 1.08	3.01 ± 1.07
Contacts		
Number of people in contact with over last 2 weeks, mean ± SD	4.39 ± 2.41	4.41 ± 2.56
Overall number of contacts (visits, phone calls) over last 2 weeks, mean ± SD	39.85 ± 31.03	46.29 ± 45.33

The WHOQOL-BREF comprises four individually scored domains. Domain scores are calculated by computing the mean scores within the domain, noting that negatively phrased questions are reverse scored. Domain scores are transformed to a 0–100 scale according to the formula in the WHOQOL Manual. Lower scores indicate a worse quality of life

*QoL* quality of life, *mMOSS* Medical Outcomes Study Social Support Survey

### Primary outcome

The greater estimated difference in slopes between treatment conditions was 3.98 points on the WHOQOL-BREF Physical subscale at week 4 (95% CI, –0.38 to 0.34). While this difference was not statistically significant, a clear trend was observed, showing that those in the immediate group did not deteriorate at the same rate as those in the wait-list group (*b* = 0.84, 95% CI, –2.24 to 3.92 vs. *b* = –3.14, 95% CI, –6.23 to –0.05) (Table 3). Furthermore, after the wait-list group received the intervention following week 4, it was observed that the difference in rate of change between groups over the follow-up period was reduced from 3.98 to 1.12 (95% CI, –2.93 to 5.19), mainly explained by a reduction in the rate of deterioration previously observed in the wait-list group (from –3.14 to –0.15; 95% CI, –2.22 to 1.92). Estimated means are presented in Table 4.

**Table 3:** Estimated rate of change from baseline to week 4 (Phase 1) and 4–12 weeks follow-up (Phase 2)

Measure	Immediate *b* (95% CI)	Wait *b* (95% CI)	Immediate vs. Wait *b* (95% CI)
*QoL Physical domain*			
Phase 1	0.84 (–2.24 to 3.92)	–3.14 (–6.23 to –0.05)	3.98 (–0.38 to 8.34)
Phase 2	0.97 (–2.51 to 4.47)	–0.15 (–2.22 to 1.92)	1.12 (–2.93 to 5.19)
*QoL Psychological domain*			
Phase 1	0.27 (–3.11 to 3.66)	–2.32 (–5.77 to 1.13)	2.59 (–2.24 to 7.43)
Phase 2	0.61 (–3.22 to 4.44)	–1.21 (–3.49 to 1.07)	1.82 (–2.63 to 6.28)
*QoL Environmental domain*			
Phase 1	–3.34 (–6.53 to –0.16)	–3.14 (–6.23 to –0.05)	0.39 (–4.13 to 4.91)
Phase 2	2.95 (–0.70 to 6.61)	0.46 (–1.69 to 2.61)	2.50 (–1.75 to 6.73)

*QoL* quality of life, *CI* confidence interval

**Table 4:** Estimated means and 95% confidence intervals at each time point for immediate and wait-list groups

Measure and time point	Immediate Estimated mean (CI)	Wait Estimated mean (CI)
*QoL Physical domain*		
Baseline	32.46 (28.99–35.92)	34.95 (31.40–38.50)
Week 4	33.29 (29.40–37.18)	31.81 (27.85–35.77)
Week 8	34.27 (30.22–38.31)	31.65 (27.96–35.35)
Week 12	35.24 (28.77–41.71)	31.50 (27.02–35.99)
*QoL Psychological domain*		
Baseline	46.60 (42.87–50.33)	46.06 (42.23–49.90)
Week 4	46.87 (42.68–51.07)	43.74 (39.44–48.04)
Week 8	47.49 (43.12–51.85)	42.53 (38.54–46.52)
Week 12	48.10 (41.04–55.15)	41.32 (36.46–46.17)
*QoL Environmental domain*		
Baseline	58.95 (55.81–62.09)	56.88 (53.64–60.12)
Week 4	55.61 (51.99–59.23)	53.15 (49.47–56.83)
Week 8	58.56 (54.74–62.38)	53.61 (50.25–56.98)
Week 12	61.52 (54.97–68.06)	54.07 (49.80–58.34)

*QoL* quality of life, *CI* confidence interval

Similar reductions from Phase 1 to Phase 2 were observed in the wait-list group when WHOQOL-BREF Psychological and Environmental subscales were primary outcomes. Per protocol analyses showed no difference to intention-to-treat analyses and were therefore not reported.

**Fig. 2:**
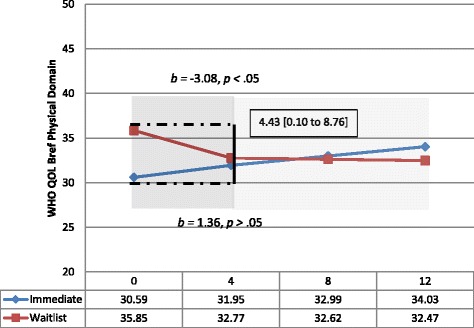
Estimated rate of change from baseline to week 4 (Phase 1) and 4–12 weeks follow-up (Phase 2) for WHOQOL-BREF Physical Domain – controlling for site and number of hours before week 4

Sensitivity analyses were conducted to take into account the amount of volunteer input people received (“number of hours of contact”) in the initial 4-week period. After controlling for site, the estimated difference in slopes between treatment conditions was 4.43 points on the WHOQOL-BREF Physical subscale at week 4 (95% CI, 0.10 to 8.76). This significant difference reflects that those in the immediate group did not deteriorate at the same rate as those in the wait-list group (Cohen d effect size = 0.27) (Table 5, Fig. 2). In terms of trajectories over the follow-up period, the pattern described above was replicated, where a reduction in the deterioration of WHOQOL-BREF subscales in the wait-list group between Phase 1 and 2 were observed.

**Table 5:** Estimated rate of change from baseline to week 4 (Phase 1) and 4–12 weeks follow-up (Phase 2) – controlling for site and number of hours before week 4

Measure and slope	Immediate *b* (95% CI)	Wait *b* (95% CI)	Immediate vs. Wait *b* (95% CI)
*QoL Physical domain*			
Phase 1	1.36 (–1.72 to 4.43)	–3.08 (–6.12 to –0.03)	4.43 (0.10 to 8.76)
Phase 2	1.04 (–2.43 to 4.51)	–0.15 (–2.19 to 1.89)	1.19 (–2.83 to 5.22)
*QoL Psychological domain*			
Phase 1	0.52 (–2.88 to 3.92)	–2.21 (–5.63 to 1.19)	2.74 (–2.08 to 7.55)
Phase 2	0.61 (–3.21 to 4.43)	–1.20 (–3.45 to 1.05)	1.81 (–2.62 to 6.24)
*QoL Environmental domain*			
Phase 1	–3.25 (–6.50 to –0.009)	–3.65 (–6.87 to –0.43)	0.40 (–4.17 to 4.96)
Phase 2	3.32 (–0.37 to 7.02)	0.46 (–1.68 to 2.61)	2.86 (–1.41 to 7.14)

*QoL* quality of life, *CI* confidence interval

### Secondary outcomes

Loneliness (De Jong Gierveld Scale [29]), social support (mMOS-SS [30]), and contacts with health and social care professionals were assessed as outcomes, where treatment effect was evaluated testing the interaction between treatment and time. No significant differences between treatment groups were found (Table 6).

**Table 6:** Loneliness, social support and reported contacts with health and social care professionals

Variables	Emotional Loneliness b	Emotional loneliness 95% CI	Social Loneliness b	Social Loneliness 95% CI	Social Support, Instrumental b	95% CI	Social Support, Emotional b	95% CI	Social Support, Total b	95% CI	Health and social care professional contact b	95% CI
Intercept	1.71	(1.43 to 1.98)	2.08	(1.85 to 2.30)	3.04	(2.75 to 3.32)	3.03	(2.80 to 3.26)	3.05	(2.81 to 3.28)	1.07	(0.77 to 1.37)
Treatment condition, Immediate	–0.20	(–0.58 to 0.17)	–0.39	(–0.71 to –0.08)^a^	0.23	(–0.17 to 0.63)	0.16	(–0.16 to 0.49)	0.19	(–0.14 to 0.52)	0.11	(–0.31 to 0.53)
Phase 1, until week 4	0.02	(–0.29 to 0.32)	0.10	(–0.17 to 0.37)	0.14	(–0.10 to 0.38)	–0.07	(–0.28 to 0.15)	0.02	(–0.16 to 0.21)	0.16	(–0.22 to 0.55)
Treatment condition × Time 1	–0.08	(–0.52 to 0.35)	–0.20	(–0.58 to 0.18)	0.02	(–0.31 to 0.36)	0.19	(–0.10 to 0.49)	0.13	(–0.13 to 0.39)	–0.21	(–0.75 to 0.34)
Phase 2, after week 4	0.02	(–0.20 to 0.23)	–0.04	(–0.22 to 0.14)	–0.08	(–0.25 to 0.09)	0.08	(–0.07 to 0.22)	0.01	(–0.12 to 0.14)	0.01	(–0.27 to 0.29)
Treatment condition × Time 2	0.07	(0.34 to 0.47)	0.22	(–0.13 to 0.57)	0.03	(–0.28 to 0.35)	–0.06	(–0.35 to 0.22)	–0.01	(–0.26 to 0.23)	–0.11	(–0.64 to 0.42)

^a^Sensitivity analyses controlling for social loneliness at baseline and week 4 did not show a different result pattern (data available upon request)

*CI* confidence interval

## Discussion

This study found no statistically significant difference in effectiveness between volunteer provided support and treatment as usual in people anticipated to be in their last year of life. We saw that the rate of reduction of quality of life, loneliness and perceived social support was less steep in the intervention group, but this did not reach statistical significance. When we controlled for the amount of volunteer support that people received we found that there was a statistically significant difference on the physical domain of quality of life, but not in other domains. There was a pattern of deteriorating levels of quality of life in the wait-list group, a decrease not observed in the immediate group, and which tends to disappear when all receive the intervention. Trends in the data are in favour of the intervention, but the effect is small, and related to reducing the rate of decline rather than improving outcomes.

### Strengths and limitations of the study

This is the first reported trial of volunteer support at the end of life, and provides important information on the outcomes of these commonly provided services, effect size and study parameters. Its particular strength is its pragmatic design, particularly as the intervention design and participants reflect the ways these services are provided and used in clinical practice rather than artificially constraining or limiting the way the intervention was provided.

Limitations include study power, blinding, missing data, attrition and intervention fidelity. Fewer service referrals were received than sites expected, although recruitment was steady over the trial period, and required numbers would have been reached with a longer recruitment period. The recruitment period was constrained by governance and contractual delays and time limited funding, which also meant alternate designs such as stepped-wedge would not be feasible [31, 32]. Our planned effect size was small, and the effectiveness of volunteer services remain unknown because of these issues with study power, and questions about choice of outcome measures. The WHOQOL-BREF was specifically chosen for its wide usage, relevance and cultural appropriateness [33–35], but there were issues with completion of the short social subscale. It may be that end of life specific quality of life scales could be appropriate and well completed [36, 37], but these were not chosen because many are disease specific, focus more on physical symptoms, flag serious illness in a way which may not be appropriate, and are not available in the same range of languages as the chosen measure. The lack of blinding could be viewed as a limitation, but it was not practical to blind patients, volunteers or site staff to treatment allocation. The amount of missing data and attrition were as expected for a study with participants at the end of life [38], and robust arrangements were in place for data cleaning and error checking (error rate 0.56%). Whilst the intervention was flexible and data were collected on its provision, not all participants received the intervention as planned. Whilst per protocol analyses revealed no difference to intention-to-treat analyses, greater fidelity in a powered trial may be important given the apparent importance of amount of volunteer input.

### Comparisons with other studies

No other trial of the outcomes of volunteer-provided support at the end of life has been conducted, although other trials are underway of volunteers building end-of-life networks [39] or supporting people with advanced dementia [40]. Previous studies of volunteer-provided services in the last year of life have been descriptive, addressing issues such as patient and volunteer experience, acceptability, facilitators and barriers [10, 11, 16, 17, 19, 41–43]. These studies have shown that volunteer provided services are seen as complementary to clinical services, and their intuitive appeal means that services are currently recommended and increasingly commonly provided by those providing a range of end-of-life care services [14].

Clinical services are known to be less accessed by those who are older, with non-malignant conditions, from ethnic minority communities or who are socio-economically disadvantaged [44–49]. Those accessing this volunteer provided service were less likely to have cancer than clinical service norms [49], and volunteer services may therefore be an access point to clinical end-of-life care services, although they did not facilitate access for those who were from black and minority ethnic communities.

Study participants had worse baseline scores for quality of life and loneliness when compared to studies of the general population or those with early stage disease [50–52], and comparable to those with similar disease stages or using clinical end of life care services [53, 54]. Together with data on deaths during the study, it is clear that those accessing a volunteer provided service are not restricted to those who are considered relatively well.

### Implications of the study for clinicians and policymakers

Clinicians often deliver palliative care within and across generalist and specialist multi-disciplinary teams, although referrals between providers can appear sparse or inequitable [44, 55, 56]. This research shows that doctors and other clinicians can confidently refer people in their last year of life to volunteer services for support which complements the clinical care offered. Clinicians should consider referring patients in the last year of life who have high social needs, and potentially those who live alone. They can expect that these services may slow a person’s decline in quality of life.

Policymakers should continue to promote the involvement of volunteers in end-of-life care. The most recent commitment from the UK government on end-of-life care emphasises community and voluntary involvement [15], building on the recognition of volunteers within the end-of-life care strategy [57], and the present research supports this policy direction. Policymakers should pay attention to making evidence-based recommendations about the amount of volunteer support provided, as a dose effect is possible.

### Future research

Further work is needed to further interrogate the effect of these interventions in powered trials, especially in determining key patient or service characteristics, and their interrelationship with services focusing on clinical needs. Important service characteristics that should be studied include the role of the volunteer as a direct supporter or in mobilising networks of support [39, 58], in the frequency and amount of support provided, and the type of person supported. Further work to determine the outcome measures best used to assess volunteer interventions is also recommended.

Research should take account of our finding that the impact is in reducing rate of decline, and rate of change should be considered as an important outcome measure in palliative and end-of-life care research.

## Conclusions

More hours or increased frequency of contact with a volunteer has a statistically significant effect on the rate of decline of physical quality of life at the end of life. Other measured outcomes of the volunteer provided support showed no statistically significant benefit over usual care, although a trend in favour of the intervention can be seen. This may have been due to the study being underpowered. This is the first trial of volunteer provided support in the last year of life and provides an emergent answer to questions of whether volunteer support should be used at the end of life; however, future trials should focus on exploring dose issues such as hours and frequency of contact as well as the type of support offered.

## Abbreviations

HLM: Hierarchical Linear Models
mMOS-SS: Medical Outcomes Study Social Support Survey
WHOQOL-BREF: World Health Organization Quality of Life Brief Scale
